# Correction: Generation of human hepatic progenitor cells with regenerative and metabolic capacities from primary hepatocytes

**DOI:** 10.7554/eLife.81959

**Published:** 2022-08-01

**Authors:** Takeshi Katsuda, Juntaro Matsuzaki, Tomoko Yamaguchi, Yasuhiro Yamada, Marta Prieto-Vila, Kazunori Hosaka, Atsuko Takeuchi, Yoshimasa Saito, Takahiro Ochiya

**Keywords:** Human, Mouse

Katsuda T, Matsuzaki J, Yamaguchi T, Yamada Y, Prieto-Vila M, Hosaka K, Takeuchi A, Saito Y, Ochiya T. 2019. Generation of human hepatic progenitor cells with regenerative and metabolic capacities from primary hepatocytes . *eLife* **8**:e47313. doi: 10.7554/eLife.47313.

Published 8 August 2019

After publication, a peer contacted us with a question regarding the method of repopulation assay using the TK-NOG mice. While replying to him, we have realized a misprinting in the Materials and methods section "Liver repopulation assay using TK-NOG mice with P0-hCLiPs". Specifically, the time period between ganciclovir injection and the following blood sampling was incorrectly described.

Corrected version: "One week after injection, approximately 30 μl blood was obtained from the tail."

Original version: "One day after injection, approximately 30 μl blood was obtained from the tail."

The article has been corrected accordingly.

